# A Polyvinyl Alcohol Hydrogel Based on a Polypyrrole/Biomass Carbon Nanosphere Synergistic Network for Flexible Pressure Sensors

**DOI:** 10.3390/gels11120956

**Published:** 2025-11-28

**Authors:** Ziyan Shu, Chunqiang Yi, Cailiu Yin, Xinjiang Zhang, Chengcheng Peng

**Affiliations:** Guangxi Key Laboratory of Advanced Structural Materials and Carbon Neutralization, Guangxi Engineering Research Center for Advanced Materials and Intelligent Manufacturing, School of Materials and Environment, Guangxi Minzu University, Nanning 530105, China

**Keywords:** carbon nanospheres, hydrogel, piezoresistive sensor

## Abstract

The rapid advancement of flexible electronics has propelled the development of lightweight, wearable piezoresistive sensors that integrate high sensitivity, excellent mechanical properties, and multifunctionality, making them a research hotspot. This work presents a flexible and lightweight multifunctional polyvinyl alcohol (PVA) composite hydrogel film, which is constructed based on a synergistic conductive network of cuttlefish ink-derived carbon nanospheres (CNPs) and polypyrrole (PPy). Within this composite, the CNPs and PPy form an interpenetrating conductive network throughout the PVA matrix, where PPy effectively suppresses the agglomeration of CNPs, thereby significantly enhancing the electron transport efficiency. This unique structure endows the material with improved flame retardancy and hydrophobicity while maintaining its lightweight characteristic. Consequently, the sensor demonstrates fast response (64 ms) and recovery times (66 ms) and a high sensitivity factor of 4.34 kPa^−1^ within a pressure range of 11.2–16.8 kPa. Excellent stability is retained after nearly 6000 loading–unloading cycles, primarily attributed to the efficient response of the contact points and conductive pathways within the synergistic network under stress. Furthermore, this flexible sensor can not only reliably monitor human physiological activities (such as finger joint bending and facial expression changes) but also generate distinct current responses to subtle mouse-clicking actions, enabling tactile handwriting input. This study provides a novel strategy for constructing high-performance sensing materials by utilizing natural biomass-derived carbon materials and conductive polymers, highlighting the significant application potential of such lightweight, multifunctional hydrogel films in next-generation flexible electronic devices.

## 1. Introduction

The rapid advancement of flexible electronics is profoundly reshaping fields such as human–machine interaction [1,2], health monitoring [3], and intelligent robotics [4,5,6], where wearable sensors, particularly piezoresistive types [7,8], have gained significant attention due to their direct mechanism of converting mechanical stress into resistance changes, alongside advantages like structural simplicity, low cost, and easy integration. Conventional flexible sensors, typically based on elastic polymers such as polyurethane or polydimethylsiloxane and incorporating conductive fillers like carbon nanomaterials or metal nanoparticles, often suffer from drawbacks including filler agglomeration, poor matrix–filler interfacial compatibility, and irreversible conductive network damage under repeated deformation, which collectively limit their overall performance [9,10,11]. Furthermore, expanding application scenarios demand enhanced lightweight characteristics, wearing comfort, and multifunctional capabilities [12], prompting research into combining conductive polymers with biocompatible hydrogels to construct stable three-dimensional interpenetrating networks [13,14]. A strategy that preserves the flexibility and biocompatibility of hydrogels while introducing excellent electrical responsiveness has been proposed, thereby opening new avenues for designing next-generation flexible sensing materials [15,16,17].

Among various hydrogel materials, polyvinyl alcohol (PVA) is regarded as an ideal matrix for constructing such composites due to its excellent biocompatibility, tunable mechanical properties, outstanding environmental stability, and well-established processing techniques [18,19]. The abundance of hydroxyl groups on its molecular chains not only imparts hydrophilic characteristics and excellent film-forming ability to the material but also provides ideal active sites for integrating diverse functional components via interactions such as hydrogen bonding. As demonstrated by Fan et al. [20], a modified double-network conductive hydrogel based on sodium alginate/PVA/black wattle tannin/carbon nanotubes exhibited high stretchability, biocompatibility, efficient self-healing performance, and considerable sensitivity. Similarly, Ding et al. [21] fabricated a PVA/sodium alginate/carbon nanotube composite hydrogel, forming a PVA/sodium alginate dual-crosslinked network alongside hydrogen bonds between carbon nanotubes and PVA, which demonstrated high stretchability (approximately 346%), good elastic recovery, and fatigue stability. Despite the increasingly extensive research available on performance optimization, there is still a lack of systematic comparative studies on the long-term performance evolution of PVA-based hydrogel sensors versus other types of hydrogel sensing systems (such as polyacrylamide-based or polyethylene glycol-based systems) under complex working conditions, as well as studies on the differences in the microscopic mechanisms of signal generation and transmission [22]. The existing literature tends to focus more on reporting the advantages of specific systems, seldom revealing the synergistic evolution mechanism between the unique hydroxyl-rich structure of PVA and the conductive network under cyclic loading and how this mechanism affects the signal stability and lifespan of sensors. These studies collectively indicate that the three-dimensional network structure formed by physical cross-linking in PVA is crucial for enhancing the interfacial bonding between fillers and the matrix and enabling effective stress transfer [23]. However, pure PVA hydrogel is inherently insulating and cannot meet the basic electrical performance requirements for sensing applications. Although the introduction of conductive fillers via physical blending represents a straightforward approach, the poor interfacial compatibility between the fillers and the hydrophilic polymer matrix often leads to phase separation and filler agglomeration during curing or under stress, resulting in an unstable conductive network and significant signal fluctuations and ultimately compromising sensor reliability and lifespan [24,25]. Therefore, constructing a uniform, stable, and durable conductive network within the PVA matrix capable of withstanding repeated deformation constitutes the core challenge in achieving high-performance sensing.

To address the aforementioned interfacial stability challenges and construct efficient composite conductive networks, researchers have explored strategies involving the synergistic combination of conductive materials with different dimensionalities. Among these, the integration of zero-dimensional carbon nanospheres (CNPs) with one-dimensional chain-like conductive polymer polypyrrole (PPy) demonstrates unique advantages. Bio-derived CNPs, characterized by their wide availability, high specific surface area, and abundant surface functional groups, offer anchoring sites that enhance binding with the polymer matrix [26,27]. PPy, renowned for its high intrinsic electrical conductivity and its ability to preferentially grow at specific interfaces via in situ polymerization, serves as an ideal conductive bridge [28,29,30]. For instance, Qin et al. [31] reported a simple and effective preparation method, successfully developing a highly tough, stretchable, and self-healing conductive hydrogel composed of PVA, Ti_3_C_2_T_x_ MXene nanosheets, and PPy. Similarly, Liu et al. [32] described a one-pot strategy for preparing hydrogels by incorporating cellulose nanofibers, PPy, and glycerol into PVA. Following PPy incorporation, the hydrogel’s electrical conductivity significantly increased to approximately 0.034 S/m, representing a 257.9% enhancement. PPy molecular chains effectively encapsulate and bridge the dispersed CNPs, thereby mitigating the agglomeration tendency of the carbon spheres and forming a continuous three-dimensional interpenetrating conductive network within the PVA matrix [33]. This structure not only enhances the electron transport efficiency of the composite material but also improves its mechanical toughness.

Based on this foundation, this study proposes a novel lightweight PVA hydrogel film incorporating a synergistic network of cuttlefish ink-derived CNPs and PPy. Utilizing cuttlefish ink as a sustainable natural carbon source, a facile, template-free process was employed to achieve the in situ polymerization of PPy within the CNP/PVA system, successfully constructing a robust CNP/PPy/PVA synergistic conductive network. This unique structure endows the composite material with high sensitivity, fast response/recovery times, and excellent stability while maintaining good flexibility. Furthermore, the developed sensor was successfully applied in the full-scale monitoring of human activities, ranging from large joint movements to subtle actions like mouse clicking and touchscreen handwriting. This work not only provides a new paradigm for developing high-performance flexible sensors using biomass-derived materials but also demonstrates their significant potential for next-generation multifunctional electronic devices.

## 2. Results and Discussion

### 2.1. Material Characterization

**Figure 1:**
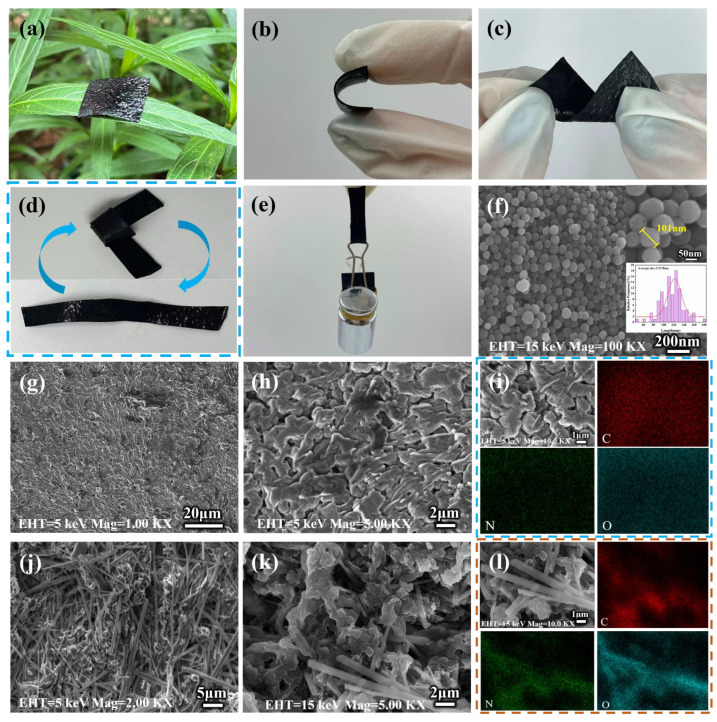
(**a**) Digital photograph of the PPy/CNP/PVA hydrogel film placed on a plant leaf. (**b**–**d**) Flexibility of the PPy/CNP/PVA hydrogel film demonstrated under bending, twisting, and knotting states, respectively. (**e**) The hydrogel film maintaining flexible morphology while supporting a 100 g weight. (**f**) SEM image of the pristine CNPs. (**g**,**h**) Surface SEM images of the PPy/CNP/PVA hydrogel film. (**i**) Corresponding C, N, and O elemental mapping on the surface of the PPy/CNP/PVA hydrogel film. (**j**,**k**) Cross-sectional SEM images of the PPy/CNP/PVA hydrogel film. (**l**) Corresponding C, N, and O elemental mapping on the cross-section of the PPy/CNP/PVA hydrogel film.

The structural characterization of the sensor determines its performance and application. Figure 1a–e visually present the macroscopic images and physical characteristics of the as-prepared PPy/CNP/PVA composite hydrogel film through digital photographs. As shown in Figure 1a, the film can be neatly placed on a plant leaf without collapsing, sufficiently demonstrating its lightweight nature. Simultaneously, the film exhibits exceptional flexibility and mechanical strength, withstanding large-scale bending (Figure 1b), twisting (Figure 1c), and knotting (Figure 1d) without fracture and recovering to its original state. Furthermore, the film maintains structural integrity even when loaded with 100 g (Figure 1e), further demonstrating its outstanding mechanical strength despite having a thickness of only 0.54 mm. This mechanical performance is attributed to the tough hydrogel network and the dynamic cross-linking interactions between PVA and glycerol molecules, which collectively enhance the mechanical strength of the composite film. Glycerol embeds itself within the PVA matrix by forming numerous hydrogen bonds with the hydroxyl groups on the PVA chains. This interaction enhances chain mobility by weakening the intermolecular forces between polymer chains. The resulting plasticizing effect endows the film with enduring flexibility and toughness, enabling it to withstand significant deformation without fracturing. Pristine CNPs display a regular nanospherical morphology (Figure 1f). Scanning electron microscopy (SEM) and energy-dispersive X-ray spectroscopy (EDS) mapping in Figure 1g reveal the surface structure of the PPy/CNP/PVA composite film at the microscopic scale, showing a relatively smooth and continuous dense network structure without obvious pores or component agglomeration. A higher magnification image (Figure 1h) further reveals that the CNPs and PPy are interwoven to form a synergistic network. This continuous network serves as the structural foundation for the film’s excellent piezoresistive sensing performance, enabling sensitive changes in electrical resistance. The corresponding EDS elemental maps for C, N, and O (Figure 1i) show a homogeneous distribution of these elements. The uniform presence of N serves as direct evidence for the successful incorporation of PPy, and the C signal originates from the conjugated carbon chains in both CNPs and PPy, while O is derived from the hydroxyl groups of the PVA matrix. The cross-sectional SEM images (Figure 1j,k) and their corresponding elemental maps (Figure 1l) further reveal a fibrous structure formed by the PVA matrix after drying, which tightly integrates with the CNP/PPy particles, creating an interpenetrating three-dimensional network. The advantage of this cross-sectional structure lies in the lightweight and flexible matrix support provided by the fibrous PVA gel network, combined with the continuous distribution of the CNP/PPy conductive network throughout the cross-section. This ensures electrical continuity in the thickness direction, thereby enhancing the sensitivity of piezoresistive sensing, and forms the microstructural basis for the material’s excellent conductivity and mechanical properties.

**Figure 2:**
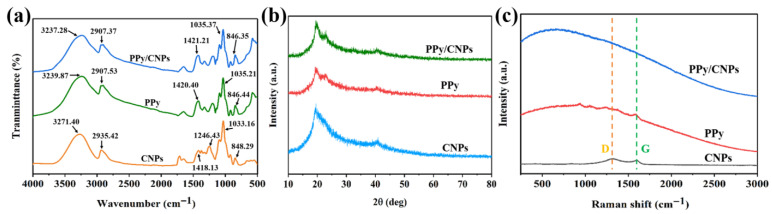
(**a**) FTIR spectra, (**b**) XRD patterns, and (**c**) Raman spectra of the CNPs, PPy, and PPy/CNP composite.

FTIR spectroscopy (Figure 2a) clearly reveals the multiple molecular interactions and synergistic effects among the components in the PPy/CNP/PVA hydrogel film. In the FTIR spectrum of CNPs, the absorption peak at 3271.40 cm^−^^1^ is assigned to N–H stretching vibration, while the peak at 1713.15 cm^−^^1^ originates from C=O stretching vibration, collectively indicating the surface functional groups rich in nitrogen and oxygen heteroatoms. The spectrum of PPy displays a characteristic N–H stretching vibration at 3239.87 cm^−^^1^ and a C=C stretching vibration within the conjugated backbone at 1651.87 cm^−^^1^, confirming its typical molecular structure. The spectrum of the PPy/CNP composite reveals distinct intermolecular interactions between the two components. The N–H stretching vibration shifts from the original positions observed in CNPs (3271.40 cm^−^^1^) and PPy (3239.87 cm^−^^1^) to a consolidated peak at 3237.28 cm^−^^1^. Concurrently, the C=C stretching vibration shifts slightly from 1651.87 cm^−^^1^ in pure PPy to 1651.30 cm^−^^1^ in the composite, accompanied by an increase in absorption intensity. These spectral changes are primarily attributed to hydrogen bonding and π–π stacking interactions formed between CNPs and PPy chains, including non-covalent interactions such as N–H and C–H. These results provide molecular-level evidence that the components are not simply physically mixed but have achieved effective interfacial bonding and chemical compatibility.

As shown in the XRD pattern in Figure 2b, the successful preparation of the PPy/CNP/PVA composite and the interactions between its components are revealed at the crystalline structure level. The CNPs exhibit a broad and weak diffuse diffraction peak at approximately 23°, corresponding to their amorphous carbon structure [34]. PPy shows a typical broad peak near 25°, indicating its non-crystalline polymer nature. After compositing, the diffraction curve of PPy/CNPs exhibits characteristics of both components, with the main diffraction peak undergoing slight shifting and shape changes. This indicates that no new crystalline phase formed during the compositing process between CNPs and PPy, preserving the non-crystalline characteristics of the individual components, while the interfacial interactions enhance the overall structural homogeneity. The predominantly disordered structure of the PPy/CNP/PVA composite is crucial for enabling the large-scale flexibility and conformability required for wearable sensing applications. These results further corroborate the uniform composite and tight coupling between the components at the nanoscale.

Raman spectroscopy (Figure 2c) provides crucial evidence regarding the carbon structure and electronic properties of the composite material. The Raman spectrum of the CNPs displays distinct D (1350 cm^−^^1^) and G (1580 cm^−^^1^) bands, with a relatively high I_D/I_G intensity ratio indicating a lower degree of structural disorder and a higher graphitization level within the carbon nanospheres [35]. The Raman spectrum of PPy, in contrast, exhibits characteristic vibration modes of its conjugated backbone. In the PPy/CNP composite, the Raman signals of both CNPs and PPy are preserved. Furthermore, discernible changes are observed in the relative intensities and positions of the D and G bands, suggesting significant charge interaction and electronic coupling between PPy and CNPs. This interfacial electronic coupling is conducive to enhancing the electron migration efficiency within the composite material, which correlates well with its improved electrical conductivity and sensing performance.

### 2.2. Properties of the Hydrogel Film

**Figure 3:**
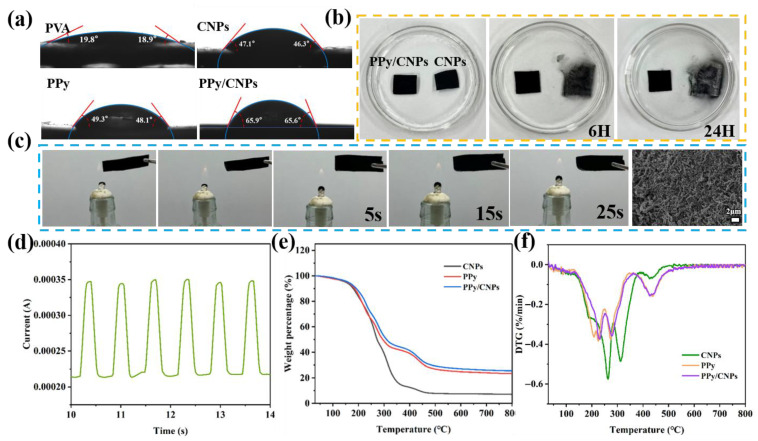
(**a**) Water contact angle test images of PVA, CNPs, PPy, and the PPy/CNP composite. (**b**) State comparison of CNPs and PPy/CNP films after being immersed in deionized water for 6 h and 24 h, respectively. (**c**) Flame retardancy test of the PPy/CNP hydrogel film and its morphology after burning in an alcohol lamp flame for 5 s, 15 s, and 25 s, respectively, with the SEM images following alcohol lamp combustion. (**d**) Sensing performance of the PPy/CNP hydrogel film after 24 h of immersion in deionized water. (**e**) TGA curves of CNPs, PPy, and the PPy/CNP composite. (**f**) DTG curves of CNPs, PPy, and the PPy/CNP composite.

Beyond electromechanical performance, practical applications of flexible electronics demand stable physicochemical properties, including hydrophobicity, flame retardancy, and environmental durability. To comprehensively evaluate the overall performance of the PPy/CNP/PVA composite film, its wettability, water stability, flame retardancy, and thermal stability were systematically investigated. The surface wettability of a material is critical for its stability in humid environments. Figure 3a shows the water contact angle (WCA) measurements for PVA, CNPs, PPy, and the PPy/CNP composite. The pure PVA film, due to its abundance of hydrophilic hydroxyl groups, is completely hydrophilic (WCA ≈ 19.8°). The pure CNP and pure PPy films show moderately increased WCAs of 47.1° and 49.3°, respectively. In contrast, the PPy/CNP composite film exhibits a significantly larger WCA (≈65.9°), demonstrating markedly enhanced hydrophobicity. This improvement is attributed to the rough micro-scale structure formed during the compositing of CNPs and PPy, which collectively enhances the material’s hydrophobic character. This hydrophobicity also contributes to excellent resistance to water erosion. As shown in Figure 3b, while a pure CNP film settled and dispersed significantly after 6 h in water and almost completely disintegrated after 24 h, the PPy/CNP composite film maintained its structural integrity without obvious collapse after 24 h of immersion. This indicates that the encapsulation by PPy effectively enhances the anchoring of CNPs within the network, substantially improving the material’s stability in aqueous environments. To verify the practical reliability of the material in humid conditions, the piezoresistive sensing performance of the PPy/CNP hydrogel film was tested after being immersed in deionized water for 24 h (Figure 3d). The results show that the post-immersion sensor could still generate clear and stable current response signals under applied pressure, with no significant degradation in its response characteristics. This robustly demonstrates the excellent stability of the synergistic conductive network constructed from PPy and CNPs in aqueous environments, ensuring reliable sensor operation in damp climates and thereby broadening its application scenarios.

Safety is a critical indicator for wearable devices. Figure 3c records the morphological changes in the PPy/CNP hydrogel film after being exposed to an alcohol lamp flame for 5, 15, and 25 s. In contrast to flammable conventional polymers, the composite film exhibits markedly improved flame retardancy. Upon brief contact with the flame, the material only undergoes localized charring without sustained combustion and rapidly self-extinguishes upon removal of the fire source. This flame retardancy originates from the ability of CNPs to form a dense char layer at high temperatures, effectively blocking oxygen and heat, while PPy likely promotes char formation and releases non-combustible nitrogen-containing gases during its decomposition, as directly evidenced by the post-combustion SEM morphology shown in Figure 3c; their synergistic action effectively interrupts the combustion cycle. Figure 3e,f present the thermogravimetric (TGA) and derivative thermogravimetric (DTG) curves of PVA, CNPs, PPy, and the PPy/CNP composite, respectively. All samples show a minor mass loss step (approximately 2–3%) near 100 °C, attributable to the evaporation of adsorbed water, a common phenomenon for hydrophilic hydrogel-based materials. In contrast to the highly hydrated, ion-rich environment of pure alginate hydrogels, which can contain >90% water and exhibit distinct water binding [36], the composite utilizes a lower water content to enhance dimensional stability and processability for flexible electronics, while the glycerol plasticizer ensures flexibility by maintaining a hydrated molecular network. The PPy/CNP composite demonstrates a residual carbon rate of 25.55% at 600 °C, significantly higher than that of pure PPy (23.41%) and CNPs (7.11%). This indicates a positive thermal synergistic effect between CNPs and PPy, where CNPs effectively promote the formation of a more stable protective char layer by PPy. For flexible wearable electronics, whose operational temperature range is closely tied to the human body (0–60 °C) and where processing temperatures rarely exceed 200 °C, the PPy/CNP/PVA composite exhibits fully sufficient, even excellent, thermal stability. It maintains most of its mass and structural integrity up to 300 °C, demonstrating its capability to withstand thermal shocks encountered during daily wear, environmental fluctuations, and manufacturing processes. The synergistic interaction between CNPs and PPy collectively endows the PPy/CNP composite with significantly enhanced thermal stability and char-forming ability compared to its individual components.

**Figure 4:**
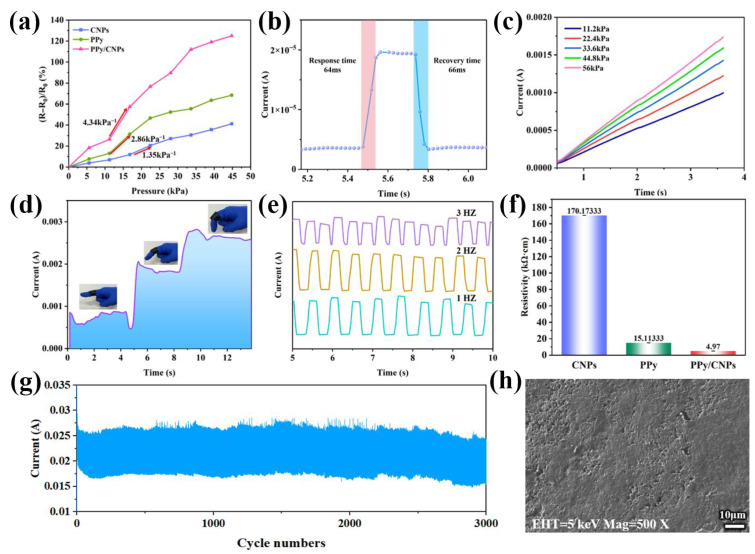
(**a**) Sensitivity comparison of PVA, CNPs, PPy, and PPy/CNP composite. (**b**) Response and recovery times of the PPy/CNP hydrogel film. (**c**) Current response of the PPy/CNP hydrogel film under pressures ranging from 11.2 kPa to 56 kPa. (**d**) Real-time current response corresponding to human finger bending at 0°, 30°, and 90°. (**e**) Comparison of current response curves for the PPy/CNP hydrogel film at 1 Hz, 2 Hz, and 3 Hz frequencies. (**f**) Resistivity measurements of PVA, CNPs, PPy, and PPy/CNP composite. (**g**) Stability test of the PPy/CNP hydrogel film under nearly 6000 loading–unloading cycles. (**h**) SEM of the PPy/CNP/PVA film surface after 6000 fatigue cycles.

To systematically evaluate the performance of the PPy/CNP/PVA composite film as a piezoresistive sensor, a series of tests were conducted on its sensitivity, response speed, resistivity, stability, and practical application capability. As shown in Figure 4a, the synergistic effect between the components is evident from the comparison of the sensitivity of PVA, CNPs, PPy, and the PPy/CNP composite. Pure PVA, being inherently insulating, exhibits very low sensitivity. The individual incorporation of CNPs or PPy introduces electrical conductivity, but their sensitivity factors remain relatively low at only 1.35 kPa^−1^ and 2.86 kPa^−1^, respectively. This limitation is likely due to the agglomeration of CNPs or the uneven distribution of PPy chains, which restricts the formation of an efficient conductive network. In contrast, the PPy/CNP composite achieves a significantly higher sensitivity factor of 4.34 kPa^−1^. This enhancement is further corroborated by resistivity measurements (Figure 4f), which show that the pure CNP film has a very high resistivity of 170.17 kΩ·cm, while the pure PPy film has a resistivity of 15.11 kΩ·cm. Remarkably, the PPy/CNP composite film demonstrates a much lower resistivity of 4.97 kΩ·cm, which is lower than that of either individual component. These results confirm that PPy acts as a conductive bridge, effectively connecting and dispersing the CNPs to form a more continuous and efficient three-dimensional conductive network. This optimized structure leads to more pronounced changes in conductive pathways under applied pressure, thereby significantly enhancing the piezoresistive performance.

The sensor also demonstrated exceptional dynamic response characteristics. As shown in Figure 4b, it exhibited rapid response and recovery times of 64 ms and 66 ms, respectively, ensuring its capability to accurately capture rapid mechanical stimuli. Table 1 presents a comparative analysis of the performance characteristics of recently reported flexible sensors based on PVA hydrogels. Among these, the PPy/CNP/PVA hydrogel pressure sensor demonstrates outstanding sensitivity and rapid response properties. Figure 4c further shows the reliable current response of the sensor across a broad pressure range of 11.2 kPa to 56 kPa, with clear and repeatable signal patterns. Furthermore, the sensor displayed excellent dynamic stability as its current signals synchronously followed cyclic loading at different frequencies (1–3 Hz) without significant attenuation (Figure 4e). Most notably, after nearly 6000 continuous loading–unloading cycles (Figure 4g), the sensor retained consistent responsiveness with no obvious signal degradation, which conclusively proves the superior mechanical robustness and structural durability of the synergistic conductive network constructed by PPy and CNPs. Figure 4h shows that after nearly 6000 continuous loading–unloading cycles, the composite film maintained its structural integrity and the interconnected conductive network without visible cracks or significant deformation, providing direct morphological evidence for the exceptional mechanical stability of the synergistic network. The practical application potential of the sensor for wearable devices was validated through a series of demonstrations. As depicted in Figure 4d, the sensor stably recorded real-time current signals corresponding to human finger bending at angles of 0°, 30°, and 90°, confirming its reliability in monitoring large-scale human joint movements. In summary, these performance results collectively verify that the composite hydrogel film, based on the PPy/CNPs synergistic network, successfully integrates high sensitivity, fast response, a broad detection range, and excellent stability, positioning it as a highly promising high-performance flexible piezoresistive sensing material.

### 2.3. Applications of Hydrogel Film Pressure Sensors

**Figure 5:**
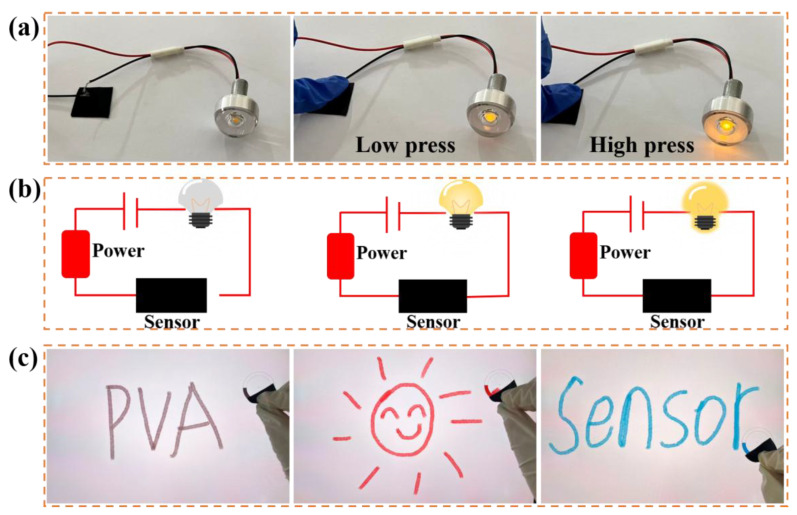
(**a**) Brightness response of a light bulb connected to the PPy/CNP hydrogel film under light and heavy pressure. (**b**) Equivalent circuit diagram of the corresponding bulb system. (**c**) Touchscreen handwriting function enabled by the PPy/CNP hydrogel film.

A series of demonstration experiments were conducted on the PPy/CNP hydrogel film to graphically illustrate its multifunctional application potential. By connecting the film in series with a light bulb to construct a visual response system (Figure 5a), a significant change in the bulb’s brightness was clearly observed upon applying different pressures to the film—the greater the pressure, the brighter the bulb. The underlying mechanism can be explained by its equivalent circuit diagram (Figure 5b): under external pressure, the number of contact points within the film’s conductive network increases and the conductive pathways become more complete, leading to a decrease in overall resistance. This reduction in resistance increases the current in the circuit, thereby driving the bulb to emit brighter light and providing direct visual validation of the material’s excellent piezoresistive response characteristics. Furthermore, the film was attached to a fingertip and successfully enabled high-precision, continuous, and smooth touchscreen handwriting input (Figure 5c). This demonstrates the material’s exceptional charge transfer capability and conformability, allowing it to effectively mimic the interaction between a human finger and a capacitive screen.

**Figure 6:**
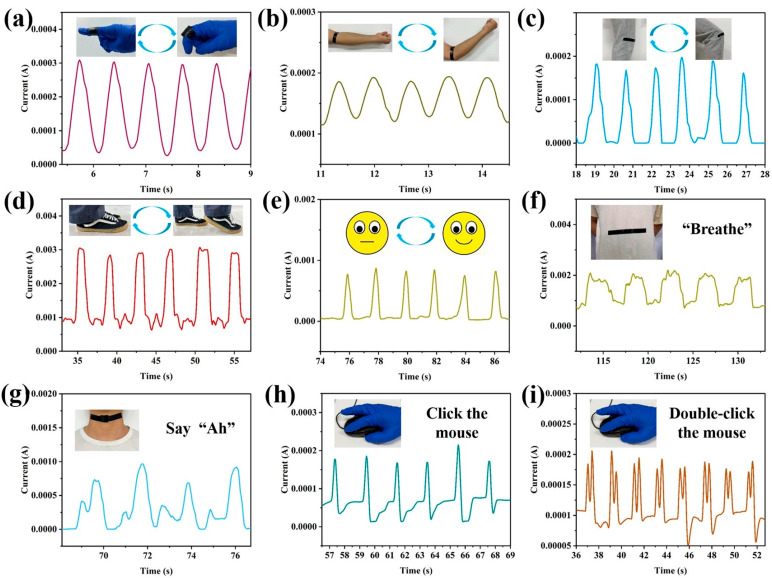
Applications of the PPy/CNP hydrogel film for human motion sensing: (**a**) finger bending, (**b**) elbow flexion, (**c**) knee bending, and (**d**) walking. Subtle motion detection: (**e**) smiling, (**f**) abdominal breathing, (**g**) vocalization (“Ah”), (**h**) mouse single-click, and (**i**) mouse double-click.

To thoroughly evaluate the performance of the PPy/CNP hydrogel film in practical wearable sensing applications, it was attached to various parts of the human body to systematically monitor activities ranging from large-scale joint movements to subtle physiological signals. As shown in Figure 6a–d, the sensor stably output clear and reproducible electrical signals corresponding to large-amplitude motions such as finger bending, elbow flexion, knee bending, and even walking, demonstrating its reliable mechanical stability and signal integrity even under substantial strain. Furthermore, the sensor accurately captured a range of very subtle physiological activities, including facial smiling, abdominal breathing, and laryngeal muscle vibrations during vocalization (Figure 6e–g). Remarkably, the sensor also generated distinctly differentiated current signals in response to mouse single-clicks and double-clicks (Figure 6h,i). This series of demonstrations conclusively shows that the PPy/CNP hydrogel film sensor successfully enables multi-scenario signal monitoring, from macroscopic limb movements to microscopic physiological activities and even external device control, highlighting its significant potential for cutting-edge applications such as human body monitoring using wearable flexible piezoresistive sensors.

## 3. Conclusions

In summary, this study successfully developed a lightweight, flexible, and multifunctional PVA composite hydrogel film for high-performance piezoresistive sensing via a simple, one-pot synthesizing and template-free preparation method. The film is based on a synergistic conductive network comprising cuttlefish ink-derived CNPs and PPy. This unique structural design endows the composite with excellent lightweight characteristics, improved flame retardancy, and enhanced hydrophobicity. The fabricated sensor demonstrates outstanding performance metrics, including a fast response time (64 ms), rapid recovery time (66 ms), and high sensitivity (4.34 kPa^−1^ within the 11.2–16.8 kPa pressure range). Notably, the sensor maintains excellent stability after nearly 6000 loading–unloading cycles, which is attributed to the efficient reorganization of contact points and conductive pathways within the CNP/PPy synergistic network under cyclic stress. Furthermore, the sensor proves to be capable of reliably monitoring various human physiological activities, detecting subtle mechanical stimuli such as mouse clicks, and enabling high-precision touchscreen handwriting input, thereby fully demonstrating its practical application potential. This work not only provides a novel and effective strategy for constructing high-performance sensing materials by leveraging the synergy between biomass-derived carbon materials and conductive polymers but also offers new design insights and a material foundation for developing next-generation wearable electronics that are environmentally friendly and integrate multiple high-performance characteristics.

## 4. Materials and Methods

### 4.1. Materials

The main experimental materials used in this study included natural cuttlefish ink collected from Xinqian Aquatic Products Company (Qingdao, China) as the precursor for deriving carbon nanospheres; pyrrole monomer (Py) (purity, 98%, CAS: 109-97-7) stored at low temperature and protected from light prior to use; polyvinyl alcohol (hydrolysis degree: 87.0–89.0%, CAS: 9002-89-5) purchased from China National Medicines Chemical Reagent Co., Ltd., China, Shanghai, ammonium persulfate (APS) (purity, 98%, CAS: 7727-54-0) as the oxidant; glycerol (purity, 99%, CAS: 56-81-5); and laboratory-prepared deionized water used throughout all experimental processes.

### 4.2. Preparation of CNPs

First, we added 50 g of cuttlefish ink to a mixture of 150 mL deionized water and 50 mL concentrated hydrochloric acid and then mechanically stirred the mixture for 24 h. Next, we centrifuged the resulting cuttlefish ink suspension at 3000 r/min for 5 min to remove larger particles. Subsequently, we centrifuged the supernatant at 9000 r/min for 10 min as one cycle. Then, we alternately washed the supernatant with deionized water and ethanol, repeating this process six times to achieve thorough purification. The resulting precipitate was dried in an 80 °C oven for 24 h. The dried solid was ground into a fine powder in a mortar, transferred to a quartz boat, and placed in a tube furnace. Under an argon atmosphere, the sample was heated at a rate of 5 °C/min to 900 °C and held at this temperature for 2 h to synthesize CNPs.

### 4.3. Preparation of PPy/CNP/PVA Composites

**Figure 7:**
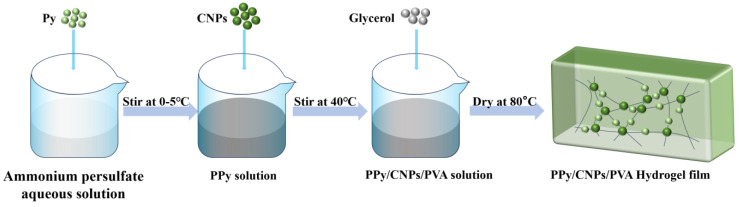
Schematic illustration of the fabrication process for the PPy/CNP/PVA composite material.

A one-pot method was employed to prepare PPy/CNP/PVA composite hydrogel films. CNPs were extracted from natural squid ink via high-temperature carbonization under an inert atmosphere. First, 0.68 g of ammonium persulfate was dissolved in 20 mL of deionized water and pre-cooled at 0–5 °C. Subsequently, 5 mL of pyrrole monomer was added to initiate oxidative polymerization. After 1 h of reaction, a suspension of carbon nanospheres (0.2 g) was introduced to form a PPy/CNP synergistic conductive network. We dissolved 2 g of polyvinyl alcohol in 20 mL of deionized water at 90 °C and then cooled the mixture to 40 °C. We added the PPy/CNPs mixture, magnetically stirred it for 30 min, then added 5 mL of glycerol and stirred for 20 min. The resulting sol–gel was dried in an 80 °C oven for 8 h to obtain a flexible, lightweight PPy/CNP/PVA composite hydrogel film. Figure 7 illustrates the detailed preparation of the PPy/CNP/PVA composite.

### 4.4. Characterization

Systematic characterization was conducted on the structure, morphology, and properties of the as-prepared cuttlefish ink-derived CNPs, PPy, and the PPy/CNP/PVA composite hydrogel film. The micro-morphology and structural features of the materials were examined using field-emission scanning electron microscopy (FE-SEM, ZEISS Gemini Sigma 300, ZEISS Oberkochen, Germany). Functional groups of the samples were analyzed with a Fourier transform infrared spectrometer (FTIR, Nicolet iS50, Thermo Fisher Scientific, Waltham, MA, USA). The carbon structure was characterized by laser confocal micro-Raman spectroscopy (Raman, Renishaw inVia, ENG, Wotton-under-Edge, Gloucestershire, UK) under 785 nm laser excitation. The crystal structure and phase composition were investigated using an X-ray diffractometer (XRD, Bruker D8 Advance, BW, Billerica, Germany). Furthermore, the thermal stability of the materials was evaluated with a Mettler TGA2 thermogravimetric analyzer under a nitrogen atmosphere from room temperature to 800 °C at a heating rate of 10 °C/min.

## Figures and Tables

**Table 1:** Performance comparison of various PVA hydrogel sensors.

Hydrogel Sensors	Sensitivity (kPa^−1^)	Response Time (ms)	Recovery Time (ms)	Ref
PPy/CNP/PVA	4.34	64	66	This work
PANF/CNT	33	160	230	[37]
MXene/PVA	0.95	-	-	[38]
PVA/SA/ENM	0.54	26.6	29.9	[39]
PVA/PAA/AG	0.121	-	-	[40]
PVA/MXOH10/hBN3	2.07	112	132	[41]

## Data Availability

The original contributions presented in this study are included in the article. Further inquiries can be directed to the corresponding author.

## Abbreviations

The following abbreviations are used in this manuscript:

PVA	Polyvinyl alcohol
PPy	Polypyrrole
CNPs	Carbon nanospheres
Py	Pyrrole monomer
APS	Ammonium persulfate
